# The Epidemiology of Neuroendocrine Carcinomas in Taiwan: A Population‐Based Cancer Registry Study

**DOI:** 10.1002/cam4.71369

**Published:** 2025-11-07

**Authors:** Yi‐Hsin Yang, Ru‐Yu Huang, Pei‐Yi Chu, Shuen‐Ru Yang, Jeng‐Shiun Du, Hui‐Jen Tsai

**Affiliations:** ^1^ National Institute of Cancer Research, National Health Research Institutes Tainan Taiwan; ^2^ Department of Pathology Show Chwan Memorial Hospital Changhua Taiwan; ^3^ Department of Post‐Baccalaureate Medicine, College of Medicine National Chung Hsing University Taichung Taiwan; ^4^ Department of Oncology National Cheng Kung University Hospital, College of Medicine, National Cheng Kung University Tainan Taiwan; ^5^ Division of Hemato‐Oncology, Department of Internal Medicine Kaohsiung Medical University Hospital, Kaohsiung Medical University Kaohsiung Taiwan

**Keywords:** epidemiology, high‐grade neuroendocrine carcinoma, nationwide, population‐based cancer registry, Taiwan, treatment patterns

## Abstract

**Background:**

Lung and small cell neuroendocrine carcinomas (SCCs) are the most common sites and histological types of high‐grade neuroendocrine carcinoma (NEC). Comprehensive epidemiological information on NECs is limited. We used the Taiwan Cancer Registry database to analyze the nationwide epidemiology and clinical outcomes of NECs in Taiwan.

**Methods:**

We used morphology codes from the International Classification of Diseases for Oncology, third edition (ICD‐O‐3) and ICD codes to identify the histologic type and sites of NECs, respectively. The Kaplan–Meier method was used to estimate the overall survival (OS) of NECs. The risk of NEC death was evaluated using Cox proportional hazards regression analysis.

**Results:**

The incidence of NECs in Taiwan was 3.892 per 100,000 in 2006 and increased to 4.039 per 100,000 in 2021, with the predominant site being the lung and bronchus and the histologic type of SCC. The median OS of all NECs was 8.3 months. Female sex, earlier stage, and later diagnosis (2016–2021) were good prognostic factors for the OS of NECs, whereas the histologic type of SCC and large cell neuroendocrine carcinoma, primary sites of the lung and bronchus, esophagus, and unknown primary sites were poor prognostic factors for the OS of NECs. Surgery combined with chemotherapy and/or radiation therapy resulted in longer survival for stage III/IV NECs.

**Conclusions:**

Differences in the incidence trends and clinical outcomes of NECs suggest different etiologies and heterogeneities of NECs. Further investigations on risk factor identification and novel treatment strategies for NECs are warranted.

## Graphical abstract

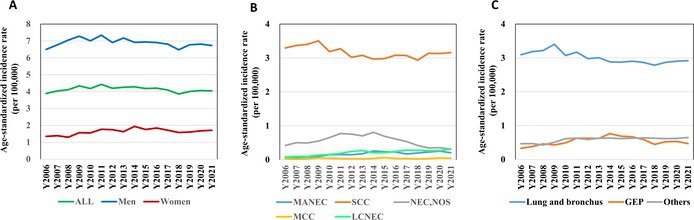

## 1 Introduction

Neuroendocrine neoplasms (NENs) are tumors with neuroendocrine differentiation that may develop throughout the whole body [1]. Pathologic classifications of NENs have been defined according to morphological changes and revised according to morphology, mitotic count, and Ki‐67 proliferation index [2, 3, 4, 5, 6]. Generally, these tumors are classified as well‐differentiated neuroendocrine tumors (NETs) or high‐grade neuroendocrine carcinomas (NECs). Recently, some tumors with high Ki‐67 but well‐differentiated morphology have been classified as grade 3 NETs [2, 7]. Many nationwide population‐based studies have reported the epidemiology of NETs [1, 8, 9, 10, 11, 12]. These studies demonstrate increasing trends in the number of NETs worldwide and different incidences in the primary site of NETs by race and geographic area [8, 9, 10, 11, 13]. In most of these studies, patients with small cell neuroendocrine carcinoma (SCC) were excluded. High‐grade neuroendocrine carcinomas include SCC, large cell neuroendocrine carcinoma (LCNEC), neuroendocrine carcinoma, NOS (NEC, NOS), and mixed adenoneuroendocrine carcinoma (MANEC). Merkel cell carcinoma (MCC) is relatively rare and has not been reported in many studies. The lung is the most common site of high‐grade NECs, with a predominance of SCCs [14, 15]. Some studies have reported on the epidemiology of LCNEC [14, 15, 16]. In addition to pulmonary NECs, some hospital‐ or population‐based studies have analyzed NECs of extrapulmonary sites, but most of them reported a specific site or a specific histological type of NEC [16, 17, 18, 19, 20, 21, 22]. For example, Brathwaite et al. have reported the characteristics, treatment, and survival of appendiceal MANEC from the SEER database [21]; Kieny et al. have reported the epidemiology of MCC in northeastern France [17]. Nationwide population‐based studies of all NECs are limited [23, 24]. In this study, we used the Taiwan Cancer Registry (TCR) database to analyze the incidence, survival, and treatment patterns of NECs in Taiwan.

## 2 Materials and Methods

### 2.1 Ethics Statement and Data Source

The study was conducted in accordance with the guidelines of the Declaration of Helsinki and approved by the Research Ethics Committee of the National Health Research Institutes, Taiwan (EC1130807‐E). This study did not require individual consent because deidentified secondary data were used for analysis. The Research Ethics Committee of the National Health Research Institutes approved the consent waiver. The study participants were extracted from the TCR and the Death Registry Database housed at the Health and Welfare Data Science Center established by the Ministry of Health and Welfare. The TCR database contains a population‐based cancer registry that includes details on cancer staging, first primary treatment, and follow‐up information to provide sufficient information for cancer control monitoring. It covers approximately 97% of cancer cases diagnosed in Taiwan [25].

### 2.2 Patient Selection

Incident cases of NECs diagnosed in Taiwan between January 1, 2006, and December 31, 2021, were identified from the TCR. Morphology codes of the International Classification of Diseases for Oncology, third edition (ICD‐O‐3) were used to identify NECs. The morphology codes used to determine the histological types of NECs included MANEC (8154, 8574), SCC (8041–8045), NEC, NOS (8246), LCNEC (8013), and MCC (8247). The ICD codes used to identify NEC sites are presented in Table S1.

### 2.3 Computation of the Annual Incidence Rate

The annual population from 2006 to 2021 reported by the Directorate‐General of Budget, Accounting, and Statistics of Taiwan (http://www.dgbas.gov.tw) was used as denominators to calculate the crude annual incidence rates. We calculated age‐standardized incidence rates by direct standardization of the age distribution of the 2000 World Health Organization standard population. The annual percentage change (APC) of NECs at each primary site was calculated using linear regression: log (rate_y_) = *b*_0_ + *b*_1_y, where log (rate_y_) = the natural log of the incidence rate in year y. APC = (e^*b*1^ − 1) × 100.

### 2.4 Statistical Analysis

Data on vital status and date of death were obtained from the death registry database to calculate overall survival (OS). The Kaplan–Meier method was used to estimate the median OS of all patients by sex, primary site, histologic type, and time period of diagnosis (T120062010: 2006–2010; T2: 2011–2015; and T3: 2016–2021). The risk of NEC death was evaluated via Cox proportional hazards regression analysis to calculate the hazard ratio (HR) and 95% confidence interval (CI) with adjusted covariates such as age, sex, primary site, histologic type, stage, and time period of diagnosis. Because TCR covers more stage information of cancer types after 2010, we analyzed only NECs diagnosed between 2011 and 2021 using the Cox model. Data management and statistical analyses were performed using SAS version 9.4 (SAS Institutes Inc., Cary, NC, USA).

## 3 Results

### 3.1 Incidence of Neuroendocrine Carcinomas (NECs) in Taiwan

A total of 23,142 cases of newly diagnosed NECs were recorded in the TCR from January 1, 2006, to December 31, 2021. The mean and median ages of all patients were 67.5 and 69.0 years, respectively (range, 6–106). The annual case numbers of all cases by sex, histologic type, and primary site from 2006 to 2021 are shown in Table S2. The male‐to‐female ratio was 3.97. The most common histological type of NEC was SCC (76.8%), followed by NEC, NOS (12.8%). The most common primary sites of NECs were the lung and bronchus (73.2%), followed by the pancreas (3.2%). Among NECs of the lung and bronchus, most patients were men, with a male (*N* = 14,993) to female (*N* = 1936) ratio of 7.74. Gastrointestinal, hepatobiliary, and pancreatic NECs are classified as gastroenteropancreatic (GEP) NECs, accounting for 12.9% of all NECs. Primary sites other than the lung and bronchus and GEP were classified as others and accounted for 13.9% of all NECs.

**FIGURE 1:**
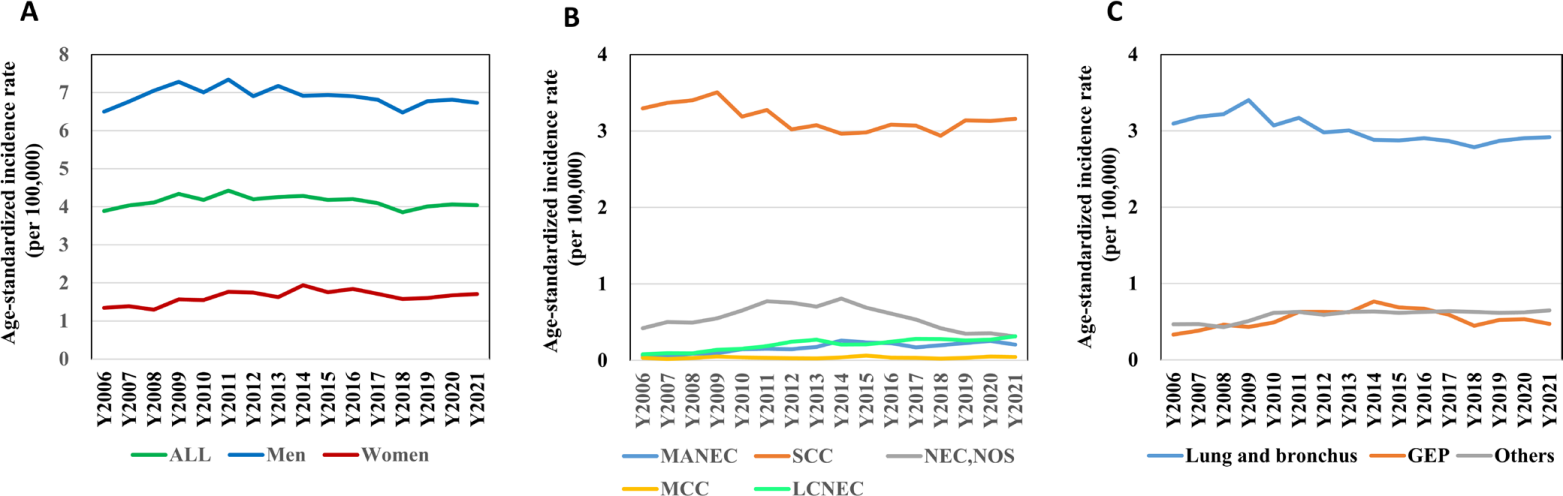
The age‐standardized incidence rate of neuroendocrine carcinomas in Taiwan from 2006 to 2021. (A) All and by sex, (B) by histologic type, (C) by 3 groups: Lung and bronchus, gastroenteropancreas (GEP), and others.

The age‐standardized annual incidence rate of NECs in Taiwan was 3.892 (6.498 in men and 1.343 in women) per 100,000 in 2006, and has increased to 4.039 (6.732 in men and 1.710 in women) per 100,000 in 2021. The annual incidence did not change significantly, with an APC of −0.162 (*p* = 0.440). In terms of histologic type, the age‐standardized incidence rates of MANEC and LCNEC increased, with APC values of 9.183 (*p* < 0.001) and 8.722 (*p* < 0.001), respectively. The annual incidence of SCC decreased, with an APC of −0.689 (*p* = 0.008). The annual incidence of NEC, NOS and MCC did not change significantly. In terms of the primary site of NECs, the annual incidence of NECs significantly increased in the colon (APC = 4.321, *p* = 0.018), female gonads (APC = 2.468, *p* = 0.016), breast (APC = 8.836, *p* < 0.001), prostate (APC = 6.827, *p* < 0.001), hepatobiliary (APC = 3.700, *p* = 0.028), bladder (APC = 7.248, *p* < 0.001) and kidney and urinary organs (APC = 7.475, *p* = 0.016). The age‐standardized incidence rates of all cases stratified by sex, histologic type, and primary site (lung and bronchus, GEP, and others) from 2006 to 2021 are shown in Table 1 and Figure 1.

**TABLE 1:** The age‐standardized incidence of NECs in Taiwan of all, by sex, histologic type and primary site.

Year	2006	2007	2008	2009	2010	2011	2012	2013	2014	2015	2016	2017	2018	2019	2020	2021	APC	*p*
All	3.892	4.038	4.110	4.341	4.180	4.426	4.197	4.255	4.282	4.180	4.205	4.093	3.860	4.011	4.062	4.039	−0.162	0.440
Men	6.498	6.762	7.048	7.284	7.008	7.341	6.905	7.172	6.917	6.935	6.903	6.811	6.477	6.776	6.810	6.732	−0.205	0.293
Women	1.343	1.390	1.297	1.567	1.553	1.768	1.743	1.626	1.938	1.754	1.842	1.712	1.578	1.603	1.673	1.710	1.408	0.015
Histologic type																		
SCC^a^, all	3.296	3.368	3.401	3.506	3.187	3.276	3.021	3.076	2.966	2.981	3.083	3.071	2.939	3.139	3.132	3.158	−0.689	0.008
Men	5.701	5.918	6.121	6.185	5.688	5.918	5.388	5.604	5.306	5.472	5.540	5.478	5.301	5.616	5.462	5.574	−0.632	0.007
Women	0.950	0.888	0.801	0.979	0.869	0.860	0.875	0.792	0.883	0.788	0.927	0.966	0.876	0.984	1.105	1.066	0.997	0.061
Pulmonary	2.961	3.036	3.085	3.190	2.844	2.923	2.660	2.679	2.558	2.610	2.653	2.570	2.460	2.611	2.632	2.646	−1.317	< 0.001
Extrapulmonary	0.335	0.332	0.316	0.0316	0.343	0.353	0.361	0.397	0.408	0.371	0.430	0.500	0.479	0.528	0.500	0.511	3.712	< 0.001
MANEC^b^, all	0.062	0.056	0.089	0.094	0.148	0.152	0.147	0.176	0.261	0.235	0.226	0.173	0.198	0.227	0.256	0.205	9.183	< 0.001
Men	0.069	0.067	0.097	0.119	0.204	0.163	0.159	0.216	0.319	0.252	0.227	0.188	0.145	0.222	0.352	0.227	8.121	< 0.001
Women	0.053	0.044	0.079	0.068	0.095	0.144	0.135	0.142	0.212	0.224	0.229	0.157	0.252	0.237	0.175	0.189	10.592	< 0.001
Pulmonary	0.014	0.010	0.004	0.013	0.034	0.036	0.034	0.024	0.037	0.050	0.027	0.035	0.043	0.027	0.042	0.026	9.351	0.008
Extrapulmonary	0.048	0.046	0.085	0.081	0.115	0.116	0.113	0.152	0.224	0.185	0.199	0.138	0.156	0.201	0.214	0.179	9.372	< 0.001
NEC, NOS^c^, all	0.422	0.501	0.495	0.551	0.653	0.774	0.756	0.705	0.808	0.690	0.613	0.534	0.421	0.348	0.353	0.315	−2.610	0.111
Men	0.555	0.613	0.651	0.675	0.817	0.931	0.913	0.913	0.910	0.833	0.686	0.687	0.578	0.446	0.490	0.377	−2.514	0.089
Women	0.288	0.394	0.347	0.438	0.497	0.637	0.614	0.519	0.722	0.558	0.551	0.401	0.278	0.259	0.233	0.263	−2.567	0.198
Pulmonary	0.076	0.074	0.067	0.096	0.086	0.112	0.120	0.122	0.150	0.087	0.067	0.067	0.095	0.067	0.063	0.055	−1.746	0.267
Extrapulmonary	0.346	0.427	0.429	0.456	0.567	0.663	0.636	0.583	0.659	0.604	0.546	0.467	0.326	0.281	0.290	0.259	−2.809	0.104
LCNEC^d^, all	0.079	0.095	0.093	0.140	0.152	0.189	0.243	0.271	0.207	0.208	0.245	0.281	0.278	0.262	0.271	0.317	8.722	< 0.001
Men	0.138	0.142	0.146	0.247	0.232	0.281	0.391	0.401	0.331	0.293	0.391	0.412	0.428	0.456	0.430	0.503	8.564	< 0.001
Women	0.020	0.049	0.041	0.041	0.078	0.104	0.109	0.155	0.092	0.135	0.114	0.163	0.149	0.090	0.132	0.152	10.689	< 0.001
Pulmonary	0.044	0.064	0.063	0.102	0.107	0.101	0.166	0.181	0.136	0.127	0.157	0.194	0.187	0.166	0.167	0.190	8.548	< 0.001
Extrapulmonary	0.035	0.031	0.030	0.038	0.045	0.088	0.077	0.090	0.071	0.081	0.088	0.087	0.091	0.096	0.104	0.126	9.268	< 0.001
MCC^e^, all	0.033	0.019	0.030	0.050	0.040	0.035	0.030	0.027	0.039	0.065	0.038	0.035	0.024	0.035	0.050	0.045	2.114	0.215
Men	0.034	0.023	0.033	0.059	0.066	0.049	0.054	0.038	0.050	0.085	0.059	0.046	0.025	0.036	0.076	0.051	2.214	0.298
Women	0.032	0.014	0.028	0.042	0.015	0.023	0.009	0.018	0.029	0.049	0.021	0.025	0.024	0.033	0.028	0.040	2.633	0.286
Extrapulmonary	0.033	0.019	0.030	0.050	0.040	0.035	0.030	0.027	0.039	0.065	0.038	0.035	0.024	0.035	0.050	0.045	2.114	0.215
Primary site																		
Lung and bronchus	3.094	3.184	3.219	3.401	3.071	3.171	2.980	3.006	2.882	2.874	2.904	2.865	2.785	2.870	2.904	2.918	−0.910	< 0.001
Men	5.580	5.781	5.908	6.191	5.715	5.866	5.490	5.610	5.379	5.457	5.513	5.409	5.286	5.408	5.439	5.461	−0.626	0.002
Women	0.665	0.655	0.644	0.767	0.614	0.707	0.700	0.652	0.648	0.596	0.611	0.630	0.595	0.658	0.691	0.699	−0.240	0.539
Small intestine	0.029	0.040	0.010	0.040	0.045	0.020	0.037	0.038	0.042	0.036	0.037	0.035	0.019	0.021	0.025	0.021	−1.208	0.603
Men	0.027	0.053	0.013	0.062	0.027	0.035	0.040	0.058	0.041	0.039	0.027	0.028	0.010	0.035	0.034	0.026	−1.923	0.479
Women	0.030	0.028	0.007	0.019	0.062	0.006	0.034	0.021	0.043	0.033	0.046	0.042	0.027	0.009	0.017	0.017	−0.225	0.945
Rectum	0.042	0.057	0.062	0.030	0.061	0.094	0.116	0.144	0.170	0.156	0.127	0.077	0.050	0.066	0.083	0.065	3.090	0.270
Men	0.042	0.095	0.087	0.025	0.080	0.124	0.122	0.187	0.212	0.217	0.149	0.084	0.091	0.090	0.109	0.106	4.653	0.136
Women	0.043	0.021	0.037	0.035	0.044	0.068	0.110	0.104	0.130	0.099	0.106	0.070	0.012	0.043	0.058	0.030	0.767	0.841
Colon	0.046	0.043	0.040	0.045	0.043	0.070	0.076	0.071	0.122	0.086	0.099	0.075	0.047	0.085	0.084	0.064	4.321	0.018
Men	0.074	0.031	0.036	0.056	0.058	0.058	0.098	0.099	0.151	0.086	0.094	0.085	0.054	0.098	0.096	0.095	5.111	0.018
Women	0.019	0.054	0.042	0.036	0.026	0.080	0.057	0.043	0.093	0.086	0.105	0.066	0.038	0.071	0.073	0.034	4.329	0.107
Stomach	0.056	0.051	0.063	0.073	0.100	0.112	0.089	0.098	0.124	0.113	0.123	0.081	0.087	0.082	0.087	0.081	2.496	0.083
Men	0.097	0.084	0.118	0.107	0.180	0.157	0.145	0.144	0.212	0.171	0.162	0.138	0.153	0.152	0.139	0.141	2.577	0.040
Women	0.013	0.018	0.013	0.042	0.027	0.070	0.038	0.059	0.047	0.064	0.088	0.031	0.029	0.021	0.044	0.032	4.566	0.155
Pancreas	0.085	0.111	0.144	0.129	0.158	0.208	0.185	0.149	0.181	0.162	0.140	0.176	0.128	0.104	0.100	0.118	−0.209	0.883
Men	0.076	0.121	0.155	0.098	0.181	0.236	0.215	0.186	0.203	0.195	0.173	0.260	0.142	0.123	0.122	0.164	1.981	0.291
Women	0.093	0.101	0.134	0.159	0.138	0.185	0.154	0.116	0.162	0.131	0.107	0.098	0.116	0.088	0.079	0.075	−2.784	0.049
Female gonads	0.237	0.265	0.150	0.213	0.228	0.251	0.266	0.281	0.273	0.203	0.311	0.311	0.243	0.263	0.305	0.330	2.460	0.016
Breast	0.038	0.047	0.014	0.031	0.047	0.040	0.066	0.059	0.088	0.071	0.082	0.080	0.133	0.100	0.061	0.083	8.836	< 0.001
Men	0.008	0.008	0.000	0.006	0.000	0.000	0.000	0.000	0.000	0.000	0.000	0.000	0.005	0.005	0.000	0.000	−4.413	0.009
Women	0.067	0.085	0.028	0.055	0.089	0.076	0.129	0.114	0.171	0.137	0.158	0.154	0.252	0.188	0.114	0.160	9.180	< 0.001
Prostate	0.027	0.057	0.031	0.052	0.036	0.084	0.071	0.086	0.070	0.077	0.070	0.079	0.076	0.111	0.094	0.079	6.827	< 0.001
Hepatobiliary	0.000	0.019	0.051	0.046	0.049	0.044	0.045	0.065	0.055	0.053	0.056	0.054	0.056	0.056	0.062	0.049	3.700	0.028
Men	0.000	0.025	0.067	0.064	0.066	0.051	0.038	0.097	0.050	0.047	0.042	0.075	0.068	0.065	0.064	0.051	2.025	0.326
Women	0.000	0.013	0.036	0.029	0.032	0.038	0.050	0.037	0.060	0.059	0.069	0.035	0.043	0.046	0.063	0.048	6.178	0.007
Esophagus	0.074	0.064	0.092	0.067	0.034	0.079	0.079	0.056	0.074	0.082	0.090	0.091	0.061	0.110	0.093	0.074	2.104	0.166
Men	0.135	0.108	0.166	0.121	0.064	0.156	0.139	0.095	0.137	0.148	0.173	0.151	0.112	0.194	0.179	0.121	1.934	0.210
Women	0.014	0.020	0.021	0.014	0.006	0.006	0.024	0.019	0.014	0.022	0.015	0.037	0.013	0.032	0.013	0.032	4.297	0.158
Head & neck	0.065	0.068	0.073	0.074	0.110	0.075	0.075	0.104	0.060	0.068	0.060	0.045	0.075	0.068	0.075	0.071	−0.917	0.422
Men	0.113	0.112	0.108	0.137	0.180	0.090	0.113	0.188	0.085	0.095	0.086	0.072	0.116	0.117	0.114	0.116	−1.172	0.410
Women	0.021	0.024	0.039	0.016	0.044	0.063	0.039	0.023	0.037	0.043	0.035	0.020	0.038	0.021	0.040	0.029	0.725	0.731
Skin	0.043	0.019	0.030	0.050	0.053	0.038	0.042	0.029	0.039	0.065	0.041	0.040	0.027	0.035	0.052	0.043	1.395	0.414
Men	0.053	0.023	0.033	0.059	0.080	0.060	0.073	0.038	0.050	0.078	0.065	0.052	0.029	0.036	0.081	0.047	1.075	0.623
Women	0.032	0.014	0.028	0.042	0.028	0.017	0.014	0.022	0.029	0.055	0.021	0.030	0.027	0.033	0.028	0.040	2.400	0.263
Bladder	0.019	0.023	0.036	0.047	0.061	0.050	0.038	0.044	0.055	0.048	0.065	0.088	0.063	0.076	0.075	0.056	7.248	< 0.001
Men	0.032	0.033	0.053	0.066	0.065	0.084	0.075	0.058	0.067	0.062	0.070	0.148	0.099	0.124	0.101	0.086	7.159	< 0.001
Women	0.007	0.013	0.019	0.029	0.056	0.018	0.006	0.032	0.045	0.035	0.062	0.036	0.030	0.035	0.052	0.031	8.711	0.018
Kidney &urinary organs	0.022	0.018	0.007	0.028	0.016	0.023	0.049	0.030	0.077	0.047	0.037	0.033	0.046	0.028	0.030	0.053	7.475	0.016
Men	0.000	0.019	0.013	0.022	0.026	0.025	0.060	0.053	0.088	0.037	0.029	0.025	0.045	0.015	0.031	0.044	3.381	0.307
Women	0.043	0.015	0.000	0.034	0.005	0.022	0.040	0.011	0.067	0.056	0.044	0.040	0.048	0.040	0.029	0.060	6.725	0.113
Thymus/mediastinum	0.035	0.025	0.047	0.054	0.068	0.083	0.039	0.077	0.045	0.036	0.033	0.048	0.036	0.035	0.045	0.041	−0.716	0.694
Men	0.050	0.043	0.057	0.069	0.107	0.110	0.072	0.117	0.047	0.048	0.060	0.044	0.052	0.052	0.074	0.051	−1.116	0.548
Women	0.020	0.008	0.036	0.040	0.032	0.056	0.008	0.038	0.043	0.025	0.010	0.052	0.020	0.017	0.020	0.032	0.467	0.897
Unknown primary	0.111	0.109	0.131	0.093	0.132	0.152	0.112	0.102	0.099	0.143	0.120	0.108	0.087	0.090	0.087	0.099	−1.651	0.075
Men	0.184	0.167	0.204	0.150	0.143	0.206	0.153	0.155	0.124	0.178	0.191	0.161	0.135	0.152	0.134	0.143	−1.476	0.069
Women	0.040	0.054	0.062	0.038	0.122	0.106	0.073	0.055	0.076	0.110	0.056	0.059	0.046	0.037	0.047	0.060	−0.914	0.663

^a^ Small cell neuroendocrine carcinoma.

^b^ Mixed adenoneuroendocrine carcinoma.

^c^ Neuroendocrine carcinoma, NOS.

^d^ Large cell neuroendocrine carcinoma.

^e^ Merkel cell carcinoma.

To understand whether the distribution of different histological types of NECs was similar between the lung and bronchus and between sites other than the lung and bronchus, we classified the sites into pulmonary (lung and bronchus) and extrapulmonary (sites other than the lung and bronchus). There are many more SCCs (2.646 per 100,000) in pulmonary NECs than LCNECs (0.190 per 100,000) and NEC, NOSs (0.055 per 100,000) in 2021. For extrapulmonary NECs, there was still more SCC (0.511 per 100,000), followed by NEC, NOS (0.259 per 100,000), MANEC (0.179 per 100,000), and LCNEC (0.126 per 100,000) in 2021. The difference in incidence among SCCs with other histological types was less significant for extrapulmonary NECs than for pulmonary NECs.

### 3.2 Percentage of Each Stage in NECs of the Lung and Bronchus, Colon, Rectum, Stomach, Esophagus, Female Gonads, Breast, Bladder, and Prostate

As only the NECs of the lung and bronchus, colon, rectum, stomach, female gonads, breast, bladder, and prostate had relatively high percentages of known stages, we analyzed these nine sites for each stage (Table 2) from 2011 to 2021. More than 50% of stage IV was in the prostate (75.76%), lung and bronchus (69.71%), and colon (53.82%). In contrast, there were more cases of stage I or II disease in NECs of the breast (75.83%). The percentages of stage III disease among these 9 NECs were similar, ranging from 16.12%–20.60%, except for breast (11.59%) and prostate (6.67%) NECs, which had lower percentages.

**TABLE 2:** The percentage of each stage in NECs of lung and bronchus, colon, rectum, stomach, esophagus, female gonads, breast, bladder, and prostate from 2011 to 2021.

Stage	I	II	III	IV	Unknown	All
Primary site	*N* (%)	*N* (%)	*N* (%)	*N* (%)	*N* (%)	*N* (%)
Lung and bronchus	400 (3.27)	230 (1.88)	2487 (20.36)	8517 (69.71)	584 (4.78)	12,218 (100)
Colon	24 (7.64)	20 (6.37)	63 (20.06)	169 (53.82)	38 (12.10)	314 (100)
Rectum^a^	126 (31.74)	9 (2.27)	64 (16.12)	113 (28.46)	85 (21.41)	397 (100)
Stomach	29 (6.87)	40 (9.48)	77 (18.25)	204 (48.34)	72 (17.06)	422 (100)
Esophagus	11 (3.37)	16 (4.91)	61 (18.71)	112 (34.36)	126 (38.65)	326 (100)
Female gonads	130 (26.21)	52 (10.48)	83 (16.73)	183 (36.90)	48 (9.68)	496 (100)
Breast	93 (30.79)	136 (45.03)	35 (11.59)	35 (11.59)	3 (0.99)	302 (100)
Bladder	29 (10.86)	77 (28.84)	55 (20.60)	75 (28.09)	31 (11.61)	267 (100)
Prostate	3 (1.82)	9 (5.45)	11 (6.67)	125 (75.76)	17 (10.30)	165 (100)

^a^ Include rectosigmoid junction and anus.

## 4 Survival of NECs

**FIGURE 2:**
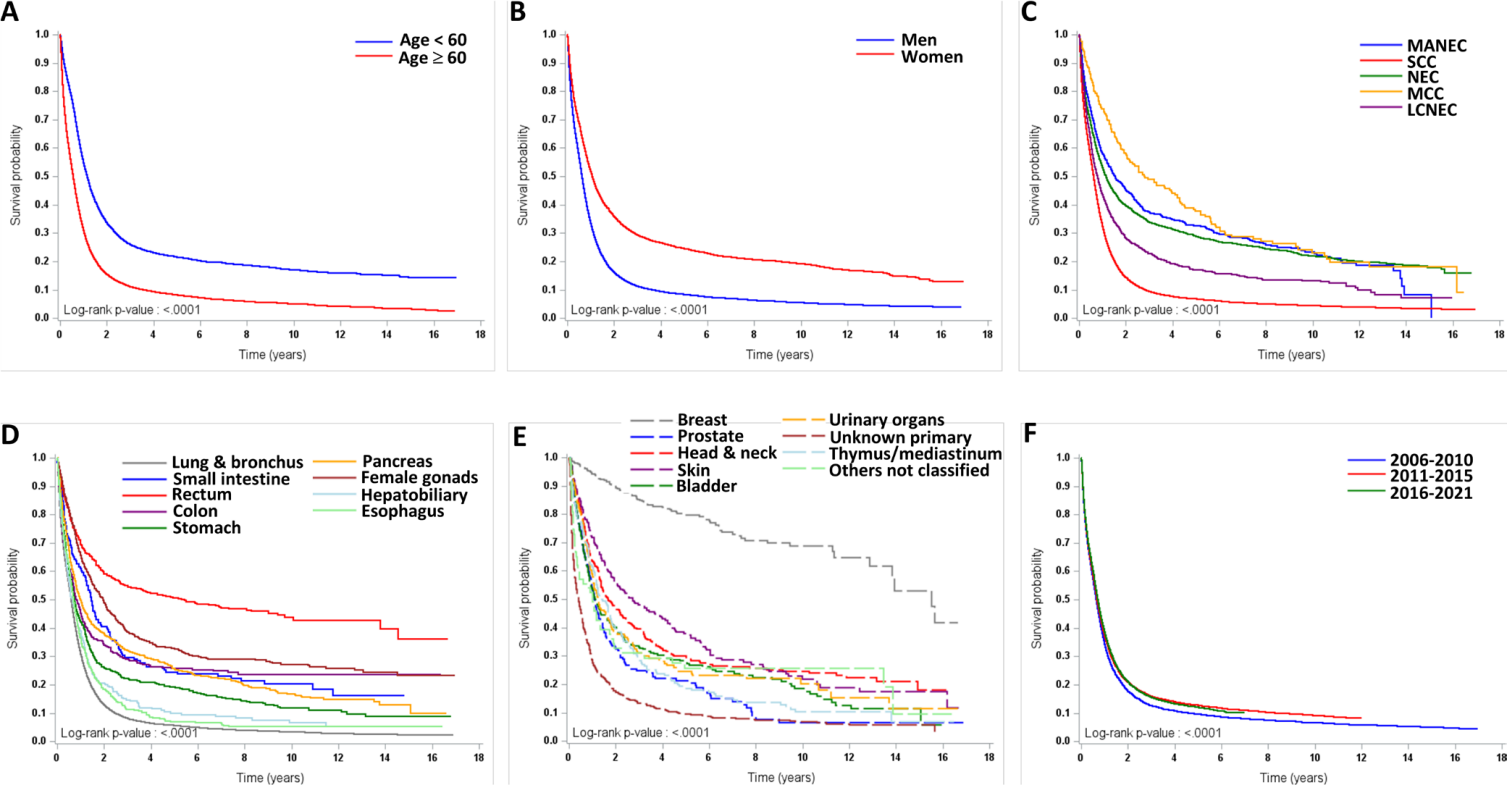
Survival curves of patients with NECs in Taiwan from 2006 to 2021. (A) Age < 60 years or ≥ 60 years, (B) sex, (C) histologic type, (D, E) primary site, (F) time period of diagnosis (2006–2010, 2011–2015, 2016–2021).

The median OS of all NECs from 2006 to 2021 was 8.3 months (95% CI, 8.1–8.4 months). The median OS and 95% CI of NECs according to sex, age, histologic type, primary site, and time period of diagnosis are shown in Table S3. Women (12.8 months) had a longer OS than men (7.6 months). Patients aged < 60 years (13.9 months) had a longer median OS than patients aged ≥ 60 years (6.8 months). In terms of histologic type, patients with MCC had the best OS (33.5 months), whereas patients with SCC (7.5 months) had the worst OS. For the primary site, patients with breast NEC (186.3 months) had the best OS. In contrast, patients with NEC of unknown primary origin (4.8 months) had the worst OS. Patients diagnosed in T3 (2016–2021) (8.6 months) had longer OS than patients diagnosed in T1 (2006–2010) (7.8 months). The survival curves of the patients according to age, sex, histologic type, primary site and time period of diagnosis are shown in Figure 2. Regarding the survival of patients with known stage information at nine sites, patients with earlier stage disease generally had better survival than those with later stage disease. However, patients with stage IV breast (34.4 months) or prostate (12.3 months) NECs had better OS than did patients with stage IV NECs at the other 7 primary sites. The median OS and 95% CI of NEC patients by stage at these nine sites are listed in Table S4.

### 4.1 Univariate and Multivariate Cox Regression Analyses of OS in NEC Patients From 2011 to 2021

We performed univariate and multivariate Cox regression analyses to evaluate the risk associated with baseline characteristics for the OS of NEC patients from 2011 to 2021, when stage information was available. According to univariate analysis, female sex, younger age, and earlier stage were good prognostic factors for OS in patients with NEC. Patients with SCC had worse survival than those with the other 4 histologic types. With respect to the primary site, only patients with NEC of the hepatobiliary region, esophagus, or unknown primary site had similar or worse survival rates than those with NEC of the lung or bronchus. Patients with NEC of primary sites other than the above three sites had better survival rates than those with NEC of the lung or bronchus (Table 3). According to multivariate analysis, female sex (HR = 0.87, 95% CI, 0.83–0.91), younger age, earlier stage, and later diagnosis were good prognostic factors for OS. With respect to histologic type, patients with MANEC (HR = 0.80, 95% CI, 0.72–0.87), MCC (HR = 0.50, 95% CI, 0.33–0.77), and NEC, NOS (HR = 0.81, 95% CI, 0.76–0.87) but not LCNEC (HR = 1.00, 95% CI, 0.93–1.08) had a lower risk for OS than patients with SCC. For primary sites, patients with NECs at most sites had a lower risk for OS than did those with NECs of the lung or bronchus. NECs of the breast (HR = 0.26, 95% CI, 0.20–0.34), kidney and other urinary organs (HR = 0.43, 95% CI, 0.36–0.52), and prostate (HR = 0.46, 95% CI, 0.38–0.54) had the lowest risk for OS.

**TABLE 3:** Univariate and multivariate Cox regression analysis for OS of NEC patients from 2011 to 2021.

*N* = 17,087		Univariate		Multivariate	
		HR	95% CI	HR	95% CI
Sex	Reference: men				
	Women	0.59	0.56–0.61	0.87	0.83–0.91
Age	Increase in 1 year	1.03	1.03–1.03	1.03	1.03–1.04
Diagnosis period	Reference: 2011–2015				
	2016–2021	0.99	0.96–1.03	0.93	0.90–0.96
Histologic type	Reference: SCC				
	MANEC	0.45	0.42–0.50	0.80	0.72–0.87
	NEC, NOS	0.51	0.49–0.54	0.81	0.76–0.87
	MCC	0.36	0.30–0.43	0.50	0.33–0.77
	LCNEC	0.70	0.65–0.75	1.00	0.93–1.08
Primary site	Reference: lung and bronchus				
	Small intestine	0.49	0.40–0.60	0.66	0.53–0.82
	Rectum^a^	0.24	0.21–0.27	0.54	0.46–0.63
	Colon	0.53	0.47–0.61	0.85	0.74–0.98
	Stomach	0.72	0.65–0.80	0.94	0.84–1.05
	Pancreas	0.58	0.52–0.63	0.82	0.73–0.92
	Female gonads	0.39	0.35–0.44	0.97	0.86–1.09
	Breast	0.08	0.06–0.10	0.26	0.20–0.34
	Prostate	0.59	0.49–0.69	0.46	0.38–0.54
	Hepatobiliary	0.88	0.77–1.02	1.10	0.95–1.28
	Esophagus	0.88	0.78–0.99	1.25	1.11–1.41
	Head and neck	0.42	0.36–0.48	0.53	0.46–0.62
	Skin	0.36	0.30–0.42	0.51	0.33–0.77
	Bladder	0.51	0.44–0.59	0.74	0.64–0.85
	Kidney & other urinary organs	0.48	0.41–0.58	0.43	0.36–0.52
	Unknown primary	1.11	1.00–1.22	1.28	1.14–1.44
	Thymus/mediastinum/others	0.58	0.49–0.68	0.76	0.64–0.91
Stage	Reference: I				
	II	1.43	1.24–1.66	1.46	1.26–1.69
	III	3.41	3.06–3.80	2.63	2.36–2.94
	IV	7.07	6.37–7.85	5.50	4.95–6.12
	Unknown	3.97	3.56–4.43	4.11	3.65–4.62

^a^ Include rectosigmoid junction and anus.

### 4.2 Proportion and Survival of NEC Patients Receiving Different Treatment Strategies

**FIGURE 3:**
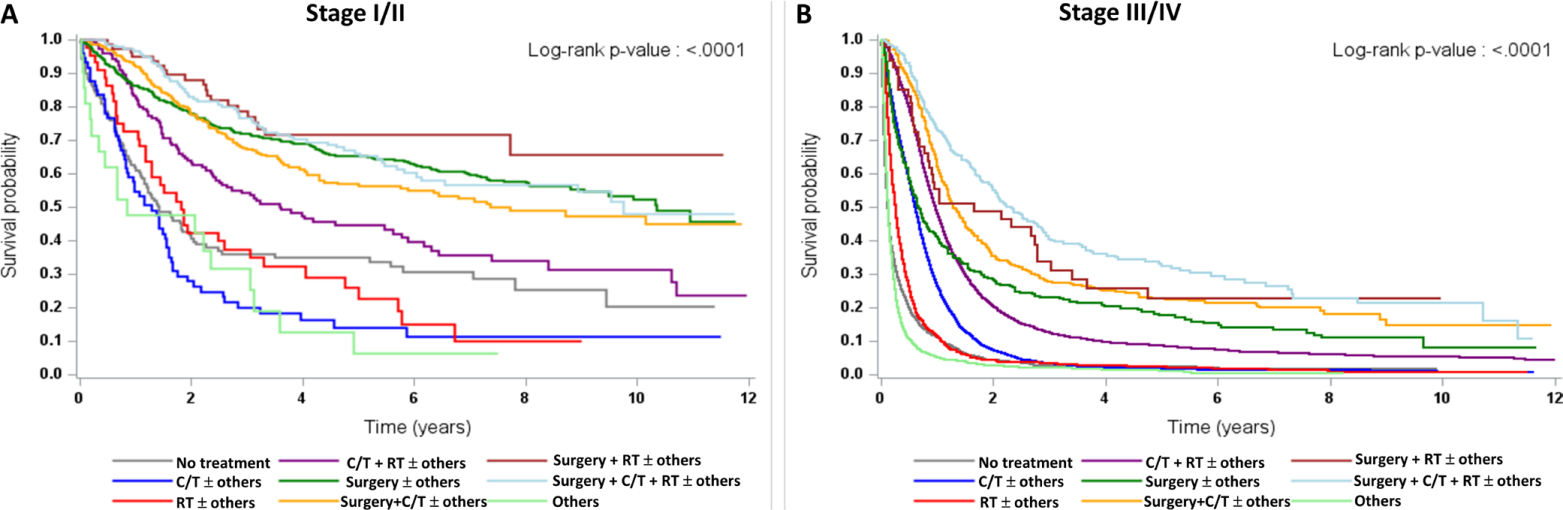
Survival curves of patients with NECs in Taiwan according to treatment strategies from 2011 to 2021. (A) Stage I/II patients and (B) stage III/IV patients.

The treatment strategies were classified as follows: surgery ± others, radiation therapy (RT) ± others, chemotherapy (C/T) ± others, surgery + RT ± others, surgery + C/T ± others, surgery + RT + C/T ± others, RT + C/T ± others, others, and no treatment. Others included hormone therapy, immunotherapy, Chinese medicine, stem cell transplantation, local C/T, local immunotherapy, targeted therapy, or other therapies not included in the above therapies. Because treatment strategies might vary according to stage, we analyzed the percentage and survival of various treatment strategies for NEC patients according to earlier stage (stage I/II) and later stage (stage III/IV) from 2011 to 2021 (Table 4). For patients with stage I/II disease, the most common treatment strategy was surgery. For patients with stage III/IV disease, the most common treatment strategy was C/T. The survival curves and median OS of the patients according to the treatment strategy and stage are shown in Figure 3 and Table S5. For stage I/II patients, the best median OS was observed for patients who received surgery + RT ± others (not reached). However, the worst median OS for stage I/II patients was observed in patients who received other treatment (10.2 months). For stage III/IV patients, the best median OS was observed for patients who received surgery + RT + C/T ± others (28.0 months). However, the worst median OS for stage III/IV patients was observed in patients who received no treatment (1.5 months).

**TABLE 4:** The percentages of treatment strategies for NECs by stage from 2011 to 2021.

	Stage I/II	Stage III/IV
	*N* (%)	*N* (%)
No treatment	121 (7.75)	1498 (11.58)
Surgery (+/− others)	553 (35.40)	276 (2.13)
RT (+/− others)	44 (2.82)	675 (5.22)
Chemotherapy (+/− others)	73 (4.67)	4015 (31.03)
Surgery + RT (+/− others)	79 (5.06)	47 (0.36)
Surgery + chemotherapy (+/− others)	331 (21.19)	414 (3.20)
Surgery + RT + chemotherapy (+/− others)	185 (11.84)	282 (2.18)
RT + chemotherapy (+/− others)	155 (9.92)	4500 (34.78)
Others	21 (1.34)	1232 (9.52)

We further analyzed the survival of three groups, namely, patients with NECs of the lung and bronchus, those with GEP, and those with other primary sites, by treatment strategy and stage (Table 5). For patients with both stage I/II and III/IV NEC of the lung and bronchus, the best median OS was for those who received surgery + RT + C/T ± others, with 114.2 months and 26.1 months, respectively. For patients with stage I/II and III/IV NEC of the GEP, the best median OS was for those who received surgery ± others (not reached, 10‐year survival rate: 68.55%) and surgery + RT + C/T ± others (20.0 months), respectively. For patients with stage I/II and III/IV NEC of other primary sites, the best median OS was for those who received surgery + RT ± others (not reached, 10‐year survival rate: 76.41%) and surgery + RT + C/T ± others (44.7 months), respectively.

**TABLE 5:** The median OS of patients with NEC of lung and bronchus, GEP and other primary sites by treatment strategy and stage from 2011 to 2021.

	Lung and bronchus				GEP				Others			
	Stage I/II		Stage III/IV		Stage I/II		Stage III/IV		Stage I/II		Stage III/IV	
	*N*	Median OS, months (95% CI)	*N*	Median OS, months (95% CI)	*N*	Median OS, months (95% CI)	*N*	Median OS, months (95% CI)	*N*	Median OS, months (95% CI)	*N*	Median OS, months (95% CI)
No treatment	48	10.7 (7.3–14.2)	1315	1.4 (1.2–1.5)	37	30.5 (13.2–NE)	102	2.4 (1.7–4.2)	36	66.8 (22.2–NE)	81	5.2 (3.9–8.4)
Surgery (+/− others)	170	50.1 (33.3–72.0)	57	10.1 (6.7–15.8)	211	NR	126	7.2 (4.7–9.6)	172	91.2 (75.3–NE)	93	7.8 (5.5–13.5)
RT (+/− others)	30	18.0 (12.7–48.6)	604	3.0 (2.6–3.3)	< 10	—	31	3.1 (2.1–3.9)	10	—	40	9.6 (5.7–13.5)
Chemotherapy (+/− others)	58	13.7 (9.7–17.7)	3600	7.4 (7.1–7.6)	< 10	—	305	6.8 (5.9–7.8)	8	—	110	7.7 (6.4–10.1)
Surgery + RT (+/− others)	5	—	6	—	< 10	—	5	—	73	NR	36	19.1 (8.3–33.5)
Surgery + chemotherapy (+/− others)	182	51.6 (42.6–88.4)	100	17.7 (14.3–22.6)	38	43.7 (26.4–NE)	199	15.4 (13.3–18.9)	111	NR	115	14.2 (11.0–19.4)
Surgery + RT + chemotherapy (+/− others)	43	114.2 (33.2–NE)	101	26.1 (20.3–34.8)	22	65.6 (21.6–NE)	64	20.0 (15.5–26.3)	120	NR	117	44.7 (27.9–86.9)
RT + chemotherapy (+/− others)	86	31.5 (21.2–47.8)	4175	11.6 (11.2–11.9)	< 10		140	8.9 (7.9–11.3)	60	77.2 (32.7–NE)	185	15.3 (13.2–19.0)
Others	8	—	1046	1.5 (1.4–1.6)	< 10		123	1.9 (1.5–2.2)	6	—	63	3.5 (2.5–6.6)

Abbreviations: ‐, not analyzable due to small sample size; NE, not evaluable; NR, not reached; RT, radiation therapy.

## 5 Discussion

This study revealed that the incidence of NECs in Taiwan was 3.892 per 100,000 in 2006 and increased to 4.039 per 100,000 in 2021, with the lung and bronchus as the predominant sites and the predominant histologic type of SCC. The incidence of NECs in men was higher than that in women, irrespective of any histological type. The median OS for all NECs from 2006 to 2021 was 8.3 months. Female sex, earlier stage, and later diagnosis (2016–2021) were good prognostic factors for the OS of NECs, whereas the histological type of SCC and LCNEC and the primary sites of the lung and bronchus, esophagus, and unknown primary sites were poor prognostic factors for the OS of NECs.

The incidence of NECs was higher in men than in women in our study, which is consistent with most studies from other countries [16, 19, 20, 22, 24, 26]. However, the incidence of pulmonary SCC in women was higher than that in men from the SEER database [14, 15]. Both genetic and environmental factors are suspected to play a role in the pathogenesis of NECs. SCC is the most common NEC in both pulmonary and extrapulmonary sites with much more in pulmonary sites. The declining trend of pulmonary SCC in Taiwan from 2006 to 2021 is consistent with the incidence of pulmonary SCC in the United States, as reported in the SEER database between 2000 and 2020 [27]. However, the incidence of extrapulmonary SCC has increased from 0.335 per 100,000 in 2006 to 0.511 per 100,000 in 2021, with an APC of 3.712 (*p* < 0.001). A decreasing incidence trend of GEP‐SCC was noted in Bavaria, Germany, according to the population‐based Bavarian Cancer Registry from 2005 to 2019 [28]. The discrepancy in the incidence trend between pulmonary and extrapulmonary SCC in Taiwan suggests different etiologies of SCC among different primary sites. In addition, the discrepancy in the incidence trends of extrapulmonary SCC between Germany and Taiwan suggests a potential racial and genetic effect on the development of SCC. Smoking is a major risk factor for pulmonary SCC, with other minor risk factors, including radon exposure; occupational exposure (asbestos, diesel engine emissions, mixtures of polycyclic aromatic hydrocarbons, crystalline silica, arsenic, and some heavy metals); and hormonal, reproductive, and dietary factors [29, 30]. The global reduction in the incidence of pulmonary SCC is consistent with a decrease in smoking prevalence [31, 32]. However, the risk factors for extrapulmonary SCC are not well understood. For the other histologic types of NECs, the increasing incidence trend of pulmonary LCNEC in Taiwan is consistent with the trend in the United States, as reported in the SEER database from 2010 to 2015 [15]. The case number of pulmonary LCNEC also increased from 2003 to 2012 in the Netherlands, according to the Netherlands Cancer Registry database [33]. These findings further suggest different etiologies and risk factors for different histological types and primary sites of NECs. In addition, several genetic and epigenetic aberrations, such as those in *TP53*, *RB1*, *NOTCH*, *ASCL1*, *DLL‐3*, *MYC*, *CREBBP*, *EP300*, *MLL1*, and *MLL2*, have been identified in pulmonary SCC [29]. *TP53* and *RB1* are commonly detected in SCCs at different primary sites. However, other genetic aberrations, such as *TERT* promoter mutations in bladder SCC, *ERG* translocations in prostate SCC, and *SMARCA4* mutations in ovarian SCC have been detected at other primary sites [34]. Genetic alterations in *TP53*, *RB1*, *STK11*, *KRAS*, *KEAP1*, *MYCL*, *SRY‐SOX2*, *PTEN*, *FGFR1*, *ASCL1*, *DLL3*, *NOTCH*, *ERBB2*, *EGFR*, and *SMARCA2* have also been reported in pulmonary LCNEC [35]. Other factors also present distinct features for specific sites of NECs, such as Merkel cell polyomavirus in skin MCC and human papillomavirus in cervical SCC [34]. These findings further suggest genetic or environmental effects on different initiation processes and tumoral characteristics of different histologic types and primary sites of NECs, and different treatment strategies for these tumors [36, 37, 38, 39, 40].

Regarding the primary site of NECs in Taiwan, the majority were pulmonary in origin, which is consistent with reports from other countries [11, 14, 15, 41]. For extrapulmonary NECs, the common incident sites vary among different countries. Data from the Netherlands Cancer Registry revealed that the most common site of extrapulmonary NECs was the bladder (28.8%) from 2008 to 2012 [24]. A report from Japan using a hospital‐based cancer registry national database revealed that the stomach (18.8%) was the most common site of extrapulmonary NECs with bladder NECs accounting for 4.8% between 2009 and 2015 [41]. In the present study, the pancreas (12.1%) was the most common site of extrapulmonary NECs from 2006 to 2021 (Table S2). Bladder NECs accounted for 5.2% of all extrapulmonary NECs cases. The difference in the distribution of common sites of NECs in different countries suggests that environmental, racial or genetic factors are responsible for the development of NECs.

In the survival analysis, we identified differential survival risk for different primary sites, particularly in stage IV patients. Patients with breast NECs (median OS = 186.3 months) had better survival than patients with other NEC sites. Although there were higher proportions of stage I/II disease (75.8%) in patients with breast NECs, the median OS of patients with stage IV breast NECs (median OS = 34.4 months) was still much better than that of stage IV patients with other sites of NECs (range of median OS, 4.9–12.3 months). The cancer‐specific survival rate at 10 years was 45.0% for breast NECs in the US from the SEER database between 2000 and 2017 [42]. The results demonstrated the distinct prognostic role of the primary site in the OS of patients with NEC.

Regarding NEC treatment strategies, we showed that surgery‐based treatment (73.5%) resulted in longer survival in patients with early‐stage disease. In contrast, C/T‐based treatments (71.2%) were the main treatment strategy for late‐stage diseases. For the largest two populations with stage III/IV disease, those with CT ± others (*N* = 4015) and those with RT + C/T ± others (*N* = 4500), those with RT + C/T ± others (median OS = 11.6 months) achieved longer survival than those with C/T ± others (median OS = 7.3 months) (Table S5). The treatment of surgery + RT + C/T ± others achieved the longest survival (median OS = 28.0 months), but the sample size was smaller (*N* = 282). Among patients with stage III/IV disease, surgery combined with RT (median OS = 19.8 months) or C/T (median OS = 15.5 months) has been shown to prolong survival. These results suggest the benefit of adding surgery to RT and/or C/T in late‐stage NEC patients, which is consistent with the improvement in survival caused by the addition of surgery in stage IV patients with other cancer types [43, 44, 45, 46]. However, patient selection based on various factors, such as better performance status, low tumor burden, and oligometastasis, may be useful for determining whether patients should undergo surgery. The performance status of patients and the availability of treatment modalities may also affect the decision on choosing a treatment strategy. These factors may affect the clinical outcome of the patients [47]. Most stage IV patients received C/T‐based treatments, and the OS of these patients was poor, except for those with breast NECs. Irinotecan plus cisplatin or etoposide plus cisplatin is the standard C/T regimen for SCC, NEC, NOS, and LCNEC at most primary sites [36, 37, 40]. The combination of cyclophosphamide, doxorubicin (or epirubicin) and vincristine is the most commonly used C/T regimen for MCC [48]. There is no standard C/T regimen for MANEC. As the median OS of patients with stage IV disease is poor, investigations of novel agents are warranted to improve survival. Recently, immunotherapy has been integrated into treatment strategies for advanced NECs and has improved patient survival [49, 50]. PD‐1 inhibitors (pembrolizumab, nivolumab, and retifanlimab) or PD‐L1 inhibitors (avelumab) have shown efficacy in disseminated MCC in prospective clinical trials [49]. PD‐L1 inhibitors (atezolizumab and durvalumab) combined with C/T have been approved as first‐line treatments for extensive pulmonary SCC, and a novel alkylating agent, lurbinectedin, has been approved as second‐line treatment for metastatic pulmonary SCC [51, 52, 53]. An antibody–drug conjugate targeting delta‐like 3 protein (DLL3), which is highly expressed in pulmonary SCC, rovalpituzumab tesirine, has been investigated for its efficacy and safety in pulmonary SCC in phase II and III clinical trials [54, 55]. DLL3‐targeted bispecific T‐cell engager tarlatamab has been investigated for its safety and efficacy in recurrent pulmonary SCC in a phase I clinical trial [56]. We expect that novel agents can be developed to improve the survival of various NECs because of advances in the understanding of the molecular biology and genetics of these tumors. Based on our findings, the clinical outcome for patients who received multiple modality treatments was better than that for those who received a single treatment strategy. The careful evaluation of patient selection for each treatment strategy and the development of novel treatment methods are important to make the best treatment for the patients.

In conclusion, this was a nationwide cancer registry‐based study that included all patients with cancer in Taiwan. The results demonstrate the heterogeneity of NECs in the epidemiology and etiology. Further understanding of the risk factors for the development of NECs and the exploration of novel treatment strategies for NECs is warranted.

## Author Contributions

**Yi‐Hsin Yang:** conceptualization (supporting), formal analysis (equal), funding acquisition (equal), investigation (supporting), methodology (equal), resources (lead), validation (equal), visualization (equal), writing – original draft (supporting), writing – review and editing (equal). **Ru‐Yu Huang:** data curation (lead), formal analysis (equal), investigation (equal), project administration (equal), software (equal), validation (equal), visualization (equal), writing – review and editing (equal). **Pei‐Yi Chu:** formal analysis (supporting), investigation (supporting), visualization (supporting), writing – review and editing (supporting). **Shuen‐Ru Yang:** formal analysis (supporting), investigation (supporting), visualization (supporting), writing – review and editing (supporting). **Jeng‐Shiun Du:** formal analysis (supporting), investigation (supporting), visualization (supporting), writing – review and editing (supporting). **Hui‐Jen Tsai:** conceptualization (lead), formal analysis (equal), funding acquisition (equal), investigation (equal), methodology (equal), supervision (lead), validation (equal), visualization (lead), writing – original draft (lead), writing – review and editing (lead).

## Disclosure

Permission to reproduce materials from other sources: All the data were generated from this study without other sources.

## Ethics Statement

The study protocol was reviewed and approved by the Institutional Review Board (IRB) of the National Health Research Institutes (EC1130807‐E). Because the analysis was based on de‐identified secondary data, individual consent was not needed.

## Conflicts of Interest

The authors declare no conflicts of interest.

## Supporting information


**Table S1:** ICD codes for identifying the sites of neuroendocrine carcinomas.

**Table S2:** The annual case number of newly diagnosed NECs in Taiwan from 2006 to 2021 of all, by sex, by histologic type, and by primary site.

**Table S3:** The median OS of NECs by age, sex, histologic type, primary site, and time period of diagnosis from 2006 to 2021.

**Table S4:** The median OS and 95% confidence interval (CI) of NEC patients by primary site and stage.

**Table S5:** the median OS of NEC patients by treatment strategy and stage from 2011 to 2021.

## Data Availability

All the data generated or analyzed during the current study is included in this published article and its Supporting Information files.
