# Supplementary material for: The Epidemiology of Neuroendocrine Carcinomas in Taiwan: A Population‐Based Cancer Registry Study
Source: Cancer Med. 2025 Nov 7;14(21):e71369. doi: 10.1002/cam4.71369 (PMC12593529; doi:10.1002/cam4.71369)
Supplement: Supplementary file 1 — Table S1: ICD codes for identifying the sites of neuroendocrine carcinomas. [file CAM4-14-e71369-s003.docx]

Supplementary Table 1. ICD codes for identifying the sites of neuroendocrine carcinomas

| Site | ICD-O-FT |
| --- | --- |
| Lung and bronchus | 162.x, 165.x |
| Small intestine | 152.x, 156.2, 159.0 |
| Rectum | 154.x |
| Colon | 153.x |
| Stomach | 151.x |
| Pancreas | 157.x |
| Female gonads | 179, 180.x, 181, 182.x, 183.x, 184.x |
| Breast | 174.x, 175.x |
| Prostate | 185 |
| Hepatobiliary | 155.x, 156.0, 156.1, 156.8, 156.9 |
| Esophagus | 150.x |
| Head and neck | 140.x-149.x, 160.x, 161.x, 195.0 |
| Skin | 173.x |
| Bladder | 188.x |
| Kidney and urinary organs | 189.x |
| Thymus/mediastinum/other | 164.x, 158.x, 159.9, 171.x, 191.1, 193, 194.x |
| Unknown primary | 199.1, 199.9 |
