# Supplementary material for: The Epidemiology of Neuroendocrine Carcinomas in Taiwan: A Population‐Based Cancer Registry Study
Source: Cancer Med. 2025 Nov 7;14(21):e71369. doi: 10.1002/cam4.71369 (PMC12593529; doi:10.1002/cam4.71369)
Supplement: Supplementary file 2 — Table S2: The annual case number of newly diagnosed NECs in Taiwan from 2006 to 2021 of all, by sex, by histologic type, and by primary site. [file CAM4-14-e71369-s004.docx]

Supplementary Table 2. the annual case number of newly diagnosed NECs in Taiwan from 2006 to 2021 of all, by sex, by histologic type and by primary site

| Year | 2006 | 2007 | 2008 | 2009 | 2010 | 2011 | 2012 | 2013 | 2014 | 2015 | 2016 | 2017 | 2018 | 2019 | 2020 | 2021 | total |
| --- | --- | --- | --- | --- | --- | --- | --- | --- | --- | --- | --- | --- | --- | --- | --- | --- | --- |
| Case number | 1077 | 1151 | 1207 | 1320 | 1300 | 1425 | 1387 | 1461 | 1511 | 1529 | 1559 | 1578 | 1537 | 1660 | 1710 | 1730 | 23142 |
| Sex | | | | | | | | | | | | | | | | | |
| Men | 889 | 953 | 1015 | 1081 | 1055 | 1139 | 1093 | 1178 | 1166 | 1197 | 1212 | 1242 | 1220 | 1325 | 1355 | 1363 | 18483 |
| Women | 188 | 198 | 192 | 239 | 245 | 286 | 294 | 283 | 345 | 332 | 347 | 336 | 317 | 335 | 355 | 367 | 4659 |
| Histologic type | | | | | | | | | | | | | | | | | |
| MANEC^a^ | 17 | 16 | 25 | 28 | 46 | 48 | 48 | 57 | 91 | 84 | 83 | 66 | 75 | 91 | 107 | 85 | 967 |
| SCC^b^ | 912 | 961 | 1003 | 1073 | 993 | 1062 | 1009 | 1063 | 1058 | 1110 | 1154 | 1193 | 1180 | 1308 | 1328 | 1368 | 17775 |
| NEC, NOS^c^ | 116 | 141 | 141 | 161 | 199 | 244 | 241 | 240 | 273 | 235 | 215 | 196 | 162 | 136 | 143 | 128 | 2971 |
| MCC^d^ | 10 | 6 | 10 | 16 | 14 | 12 | 11 | 10 | 16 | 26 | 17 | 14 | 11 | 17 | 24 | 23 | 237 |
| LCNEC^e^ | 22 | 27 | 28 | 42 | 48 | 59 | 78 | 91 | 73 | 74 | 90 | 109 | 109 | 108 | 108 | 126 | 1192 |
| Primary site | | | | | | | | | | | | | | | | | |
| Lung and bronchus | 856 | 908 | 951 | 1040 | 956 | 1029 | 993 | 1043 | 1035 | 1071 | 1089 | 1118 | 1125 | 1207 | 1242 | 1266 | 16929 |
| Small intestine | 8 | 11 | 3 | 12 | 13 | 6 | 12 | 13 | 15 | 14 | 13 | 14 | 8 | 9 | 11 | 9 | 171 |
| Rectum | 11 | 16 | 18 | 9 | 20 | 30 | 37 | 48 | 55 | 50 | 43 | 29 | 20 | 27 | 31 | 27 | 471 |
| Colon | 13 | 12 | 11 | 13 | 14 | 23 | 23 | 24 | 39 | 30 | 35 | 27 | 18 | 34 | 34 | 27 | 377 |
| Stomach | 17 | 15 | 19 | 22 | 34 | 38 | 31 | 35 | 45 | 43 | 47 | 34 | 38 | 34 | 40 | 37 | 529 |
| Pancreas | 22 | 31 | 40 | 38 | 48 | 62 | 59 | 48 | 61 | 53 | 49 | 61 | 49 | 39 | 39 | 50 | 749 |
| Female gonads | 33 | 38 | 21 | 32 | 35 | 39 | 43 | 43 | 44 | 34 | 51 | 53 | 39 | 47 | 49 | 54 | 655 |
| Breast | 10 | 13 | 4 | 9 | 14 | 12 | 20 | 19 | 28 | 25 | 28 | 29 | 48 | 37 | 25 | 31 | 352 |
| Prostate | 4 | 8 | 5 | 7 | 6 | 13 | 11 | 14 | 12 | 13 | 12 | 16 | 15 | 23 | 19 | 17 | 195 |
| Hepatobiliary | 0 | 5 | 15 | 14 | 15 | 16 | 15 | 23 | 19 | 18 | 21 | 21 | 22 | 23 | 27 | 22 | 276 |
| Esophagus | 20 | 18 | 26 | 20 | 11 | 24 | 26 | 20 | 25 | 28 | 35 | 34 | 23 | 44 | 36 | 31 | 421 |
| Head and neck | 17 | 19 | 21 | 21 | 32 | 23 | 24 | 34 | 20 | 24 | 22 | 17 | 27 | 23 | 29 | 28 | 381 |
| Skin | 13 | 6 | 10 | 16 | 18 | 13 | 15 | 11 | 16 | 27 | 18 | 16 | 13 | 17 | 25 | 22 | 256 |
| Bladder | 6 | 7 | 11 | 15 | 20 | 16 | 13 | 16 | 21 | 18 | 25 | 36 | 28 | 33 | 34 | 27 | 326 |
| Kidney and urinary organ | 6 | 5 | 2 | 8 | 5 | 8 | 16 | 10 | 26 | 17 | 14 | 14 | 18 | 12 | 13 | 23 | 197 |
| Thymus, mediastinum and others | 10 | 7 | 13 | 16 | 20 | 24 | 12 | 24 | 15 | 13 | 13 | 18 | 12 | 13 | 18 | 16 | 244 |
| Unknown primary | 31 | 32 | 37 | 28 | 39 | 49 | 37 | 36 | 35 | 51 | 44 | 41 | 34 | 38 | 38 | 43 | 613 |

a, mixed adenoneuroendocrine carcinoma; b, small cell neuroendocrine carcinoma; c, neuroendocrine carcinoma, NOS; d, Merkel cell carcinoma; e, large cell neuroendocrine carcinoma.
