# Supplementary material for: The Epidemiology of Neuroendocrine Carcinomas in Taiwan: A Population‐Based Cancer Registry Study
Source: Cancer Med. 2025 Nov 7;14(21):e71369. doi: 10.1002/cam4.71369 (PMC12593529; doi:10.1002/cam4.71369)
Supplement: Supplementary file 3 — Table S3: The median OS of NECs by age, sex, histologic type, primary site, and time period of diagnosis from 2006 to 2021. [file CAM4-14-e71369-s002.docx]

Supplementary Table 3. The median OS of NECs by age, sex, histologic type, primary site and time period of diagnosis from 2006 to 2021.

| Variable | Median OS (months) | 95% CI  (months) |
| --- | --- | --- |
| All | 8.3 | 8.1-8.4 |
| Age | | |
| < 60 | 13.9 | 13.5-14.4 |
| ≥ 60 | 6.8 | 6.6-7.0 |
| Sex | | |
| Men | 7.6 | 7.5-7.8 |
| Women | 12.8 | 12.2-13.5 |
| Histologic type | | |
| MANEC^a^ | 18.4 | 15.8-22.4 |
| SCC^b^ | 7.5 | 7.4-7.7 |
| NEC, NOS^c^ | 13.8 | 12.8-14.9 |
| MCC^d^ | 33.5 | 24.9-49.4 |
| LCNEC^e^ | 9.6 | 8.4-10.5 |
| Primary site | | |
| Lung and bronchus | 7.2 | 7.1-7.4 |
| Small intestine | 17.6 | 14.7-20.9 |
| Rectum* | 65.1 | 36.3-108.4 |
| Colon | 10.1 | 8.4-12.2 |
| Stomach | 8.8 | 7.6-10.0 |
| Pancreas | 12.1 | 10.2-13.7 |
| Female gonads | 23.5 | 19.3-26.3 |
| Breast | 186.3 | 165.8-NE |
| Prostate | 13.0 | 11.0-15.2 |
| Hepatobiliary | 8.0 | 6.3-9.6 |
| Esophagus | 8.3 | 7.4-9.5 |
| Head and neck | 20.1 | 16.4-26.2 |
| Skin | 32.6 | 24.0-47.2 |
| Bladder | 13.5 | 11.1-16.4 |
| Kidney & urinary organs | 14.7 | 12.5-20.0 |
| Thymus/mediastinum/others | 14.7 | 12.9-20.3 |
| Unknown primary | 4.8 | 3.7-5.9 |
| Diagnosis year | | |
| T1:2006-2010 | 7.8 | 7.6-8.1 |
| T2:2011-2015 | 8.2 | 8.0-8.4 |
| T3:2016-2021 | 8.6 | 8.4-8.9 |

a, mixed adenoneuroendocrine carcinoma; b, small cell neuroendocrine carcinoma; c, neuroendocrine carcinoma, NOS; d, Merkel cell carcinoma; e, large cell neuroendocrine carcinoma.

*, include rectosigmoid junction and anus
