# Supplementary material for: The Epidemiology of Neuroendocrine Carcinomas in Taiwan: A Population‐Based Cancer Registry Study
Source: Cancer Med. 2025 Nov 7;14(21):e71369. doi: 10.1002/cam4.71369 (PMC12593529; doi:10.1002/cam4.71369)
Supplement: Supplementary file 4 — Table S4: The median OS and 95% confidence interval (CI) of NEC patients by primary site and stage. [file CAM4-14-e71369-s001.docx]

Supplementary Table 4. The median OS and 95 % confidence interval (CI) of NEC patients by primary site and stage

|  | Lung and bronchus | Rectum | Colon | Stomach | Female gonads | Breast | Esophagus | Bladder | Prostate |
| --- | --- | --- | --- | --- | --- | --- | --- | --- | --- |
| Stage | Median OS, months  (95% CI) | Median OS, months  (95% CI) | Median OS, months  (95% CI) | Median OS, months  (95% CI) | Median OS, months  (95% CI) | Median OS, months  (95% CI) | Median OS, months  (95% CI) | Median OS, months  (95% CI) | Median OS, months  (95% CI) |
| I | 38.5  (31.0-48.3) | NR** | NR** | 69.5  (21.7-NE*) | NR** | NR** | 18.4  (9.4-NE*) | 61.7  (30.1-NE*) | NA# |
| II | 23.8  (19.8-33.2) | NR** | 87.4  (6.3-NE*) | 65.6  (22.6-NE*) | 50.0  (33.9-128.4) | NR** | 40.3  (21.6- NE*) | 28.2  (19.5-60.3) | NA# |
| III | 12.9  (12.3-13.5) | 24.3  (16.2-45.5) | 92.6  (17.5-NE*) | 14.8  (10.5-18.8) | 21.6  (13.5-28.3) | 86.9  (34.8-NE*) | 11.3  (8.7-15.5) | 21.6  (8.8-32.9) | 91.0  (8.0-NE*) |
| IV | 5.9  (5.7-6.1) | 6.5  (4.2-8.0) | 4.9  (3.5-6.8) | 4.9  (4.1-6.00) | 8.9  (7.3-10.4) | 34.4  (14.3-72.2) | 5.9  (3.4-6.8) | 6.8  (4.4-8.3) | 12.3  (10.0-15.2) |

*, NE: not evaluable

**, NR: not reached

#, NA: not analyzable due to small sample size
