# Supplementary material for: The Epidemiology of Neuroendocrine Carcinomas in Taiwan: A Population‐Based Cancer Registry Study
Source: Cancer Med. 2025 Nov 7;14(21):e71369. doi: 10.1002/cam4.71369 (PMC12593529; doi:10.1002/cam4.71369)
Supplement: Supplementary file 5 — Table S5: the median OS of NEC patients by treatment strategy and stage from 2011 to 2021. [file CAM4-14-e71369-s005.docx]

Supplementary Table 5. the median OS of NEC patients by treatment strategy and stage from 2011 to 2021.

|  | Stage I/II | Stage III/IV |
| --- | --- | --- |
|  | Median OS, months (95% CI) | Median OS, months (95% CI) |
| No treatment | 16.9 (12.2-23.8) | 1.5 (1.4-1.6) |
| Surgery (+/- others) | 124.2 (108.4-NE*) | 8.0 (6.7-10.0) |
| RT (+/- others) | 21.8 (14.0-36.7) | 3.2 (2.9-3.4) |
| Chemotherapy (+/- others) | 15.5 (9.9-18.5) | 7.3 (7.1-7.6) |
| Surgery + RT (+/- others) | NR** | 19.8 (10.1-33.5) |
| Surgery + chemotherapy (+/- others) | 88.4 (68.9-NE*) | 15.5 (14.3-18.0) |
| Surgery + RT + chemotherapy(+/- others) | 117.0 (73.5-NE*) | 28.0 (23.7-34.3) |
| RT + chemotherapy (+/- others) | 43.3 (29.1-69.2) | 11.6 (11.3-11.9) |
| Others | 10.2 (2.3-36.7) | 1.6 (2.5-1.7) |

*, NE: not evaluable

**, NR: not reached
